# Maternal socioeconomic factors and the risk of premature birth and low birth weight in Cyprus: a case–control study

**DOI:** 10.1186/s12978-018-0603-7

**Published:** 2018-09-19

**Authors:** Paraskevi Stylianou-Riga, Panayiotis Kouis, Paraskevi Kinni, Angelos Rigas, Thalia Papadouri, Panayiotis K. Yiallouros, Mamas Theodorou

**Affiliations:** 1 Neonatal Intensive Care Unit, “Archbishop Makarios III” Hospital, Nicosia, Cyprus; 2 Medical School, University of Cyprus Shakolas Educational Center of Clinical Medicine, Palaios Dromos Lefkosias Lemesou 215/6, 2029 Aglantzia, Nicosia, Cyprus; 3 Apollonion Hospital, Nicosia, Cyprus; 4 Faculty of Economics and Management, Open University of Cyprus, Nicosia, Cyprus

**Keywords:** Preterm birth, Low birth weight, Maternal risk factors, Socioeconomic factors

## Abstract

**Background:**

Prematurity and low birth weight are significant predictors of perinatal morbidity and mortality and are influenced by the overall health and socioeconomic status of the pregnant mother. Although Cyprus is characterized by the highest prematurity rate in Europe (13.1% in 2014), the relationship between maternal health and socioeconomic characteristics with prematurity and low birth weight has never been investigated. We aimed to investigate the association of maternal demographic, clinical and socioeconomic characteristics with premature delivery and low neonatal birth weight in Cyprus.

**Methods:**

In a case-control design, questionnaire data were collected from 348 women who gave birth prematurely (cases) and 349 women who gave birth at term (controls). Information was obtained on gestation duration and birth weight as well as maternal demographic, socioeconomic and clinical profiles, including parameters such as smoking, body mass index, alcohol consumption, presence of gestational diabetes and mental health factors.

**Results:**

Premature delivery was associated with greater maternal age (OR: 1.12, 95% CI: 1.06–1.18), absence of gestational diabetes (OR: 0.53, 95% CI: 0.30–0.97), long working hours (OR: 3.77, 95% CI: 2.08–6.84) and emotional stress (OR: 8.5, 95% CI: 3.03–23.89). Within the cases group, emotional stress was also associated with lower birth-weight (β: -323.68 (95% CI: -570.36, − 77.00).

**Conclusions:**

The findings of this study demonstrate the positive association of maternal psychological factors, working conditions as well as maternal age with prematurity and low birth weight in Cyprus. Additional, prospective, studies are needed in the country to further investigate these associations and inform public health intervention measures.

**Electronic supplementary material:**

The online version of this article (10.1186/s12978-018-0603-7) contains supplementary material, which is available to authorized users.

## Plain english summary

Prematurity and low birth weight are important determinants of neonatal health and are influenced by the overall health and socioeconomic conditions of the pregnant mother. While prematurity rates vary significantly across the world, Cyprus is characterized by the highest prematurity rate in Europe. However, the factors that are associated with prematurity and low birth weight in Cyprus have never been studied.

This study was designed to understand the influence of demographic, mental health and socioeconomic factors on premature delivery and low birth weight in Cyprus. We identified 348 mothers with premature deliveries at the island’s largest maternity unit during a one year period and we also enrolled 349 mothers with term pregnancies as controls. Through the use of a specifically designed questionnaire we obtained data on socioeconomic characteristics from both groups of mothers. Basic information about clinical and demographic characteristics was available from medical charts.

Comparison between mothers with preterm pregnancies and mothers with term pregnancies demonstrated that maternal age at birth, long working hours and emotional stress were associated with a higher risk for prematurity while emotional stress was also associated with lower birth weight among premature neonates. Combinations of two or more factors were associated with a sharp increase of prematurity risk, demonstrating a cumulative effect.

Healthcare professionals should take into account the overall socioeconomic status of pregnant women during observation, towards identifying high risk pregnancies for prematurity and implementation of appropriate clinical management. Furthermore, healthcare policy makers should aim for the development of public health intervention measures targeting high risk pregnant women.

## Background

Optimal fetal development is widely recognized (World Health Organization – WHO, 2006) as an important factor of infant’s survival and subsequent social development. In particular, birth weight, neonatal viability and gestational age, are considered as important health determinants throughout lifetime [1]. Furthermore, the good health and the favorable socioeconomic environment of the pregnant mother are also considered essential prerequisites for the mental and physical well-being of the infant [2].

Preterm labor is the leading cause of perinatal morbidity and mortality in developed countries, where the majority of deaths occur in neonates with a gestational age of less than 32 weeks [3–5]. In recent years, the care provided in Neonate Intensive Care Units (NICU) settings increased the survival of premature infants but at the same time increased duration of hospitalization and costs. As a result, the care of premature neonates currently accounts for a large proportion of the total in-hospital costs worldwide [6]. In total, fifteen million premature births are reported annually worldwide [4] and although the frequency of preterm labor varies considerably between countries, almost 90% of these premature births occur in developing countries in Africa and Asia [7]. In 2014, the rate of preterm births was 10% in the US [8], while in Europe in 2010, preterm birth rates varied markedly from 5 to 10.6% among live births [9]. Cyprus is characterized by the highest premature birth rate in Europe, reaching 10.6% and 13.1% in 2010 and 2014 respectively [10, 11] partly due to increase of multiple pregnancies following in-vitro fertilization [12].

Furthermore, low birth weight (LBW) is associated with increased mortality as well as acute and long-term health problems [13–15]. In Europe the percentage of LBW ranged between 4 and 9% in 2010 [10]. LBW infants also face significant neurodevelopmental problems, whereas in adulthood they are at higher risk to develop type 2 diabetes mellitus, hypertension and coronary artery disease [16].

Several risk factors have been found to be associated with premature birth and LBW, including maternal age, ethnicity, lifestyle maternal characteristics such as smoking and alcohol consumption, education level, working conditions, access to obstetric observation, diabetes mellitus, mental stress and depression, body mass index (BMI) before pregnancy and additional weight-gain during pregnancy [5]. Despite the increasing incidence of premature births in Cyprus, there is lack of knowledge regarding the prevalent maternal risk factors for premature birth and LBW, which is a necessary prerequisite for development of prevention strategies by the national health system and awareness initiatives among the public and health professionals. We aimed to assess the relation of demographic and maternal socioeconomic and lifestyle characteristics with the risk of premature birth among Cypriot pregnant women and investigate the contribution of the same characteristics to LBW risk among premature infants.

## Methods

### Study population

The source population were all mothers that gave birth between March 2015 and April 2016 at “Archbishop Makarios III” hospital in Nicosia, Cyprus. “Archibishop Makarios III” hospital is the main tertiary maternal and pediatric hospital in Cyprus which hosts the only tertiary NICU on the island. Mothers who gave birth to premature neonates (gestation < 37 weeks) were included in the study as cases and mothers that gave birth to term neonates (gestation > 37 weeks) were included in the study as controls. Data collection commenced in March 2015 and was completed in April 2016. Mothers with multiple pregnancies or mothers that underwent infertility treatment as well as mothers of stillborn neonates and mothers of neonates with chromosomal abnormalities were excluded from the study. Sample size calculation was performed using OpenEpi [17] assuming a 1:1 ratio of cases to controls, 95% confidence level, 80% power and a least extreme Odds Ratio (OR) to be detected equal to 1.55. The calculated sample size was 333 cases and 333 controls. All participants provided written informed consent and the study protocol was approved by the Cyprus National Bioethics Committee (EEBK EΠ 2015.01.25) and the Cyprus Ministry of Health (Protocol approval: 0282/2015).

### Data collection and data processing

Information on duration of gestation period and birth weight was obtained from hospital records while all other information was collected through a structured self-administered questionnaire. The questionnaire (Additional file 1) was adopted from similar previous studies [18, 19] and consisted of questions on basic demographic (age, ethnicity) and socioeconomic factors such as marital status, socioeconomic status (income, education level, profession, working conditions (manual labour, standing, > 8 h shifts). Data on anthropometric indices and other potential maternal risk factors such as gestational diabetes, stress, depression, anti-depressants consumption, alcohol consumption, and smoking before and during pregnancy were also obtained through the questionnaire and the responses were crosschecked with available information in the mothers’ medical records. Anthropometric and clinical parameters such as weight, height, gestational diabetes and depression were derived from clinical assessments while experience of emotional stress and use of antidepressants or anxiolytics during pregnancy were obtained from self-assessment by the participants using binary (YES or NO) questions. Composite scores for variables describing working conditions and socioeconomic deprivation were developed with individual factors carrying the same weight.

### Statistical analysis

All variables used in the analysis were checked for normality with the use of histograms. Summary statistics for participant characteristics were calculated separately for cases and controls and categorical variables are reported as absolute and relative (%) frequencies, while continuous variables are reported as mean estimates and standard deviation. In unadjusted analyses, differences in participant characteristics between cases and controls were investigated using chi square test in the case of categorical variables and independent *t*-test in the case of continuous variables. A multivariate logistic regression including all variables that were found to be significantly associated with prematurity (*p*_value_ < 0.10) in the unadjusted analysis was used in order to calculate the OR and 95% Confidence Intervals (95% CI) for pre-term birth.

Among cases, the relationship between different characteristics and gestational age as well as birth weight were examined using the Pearson correlation coefficient and adjusted analyses were performed using a multivariate linear regression model. The linear regression models included all variables that were significantly correlated with birth weight (*p*_value_ < 0.10).

To further illuminate the role of clustering of risk factors for prematurity, a cumulative individual risk score was calculated for each mother by summing the values (0 for negative or 1 for positive) of each significant risk factor. Lastly, to demonstrate the extent to which different significant risk factors overlap, a Venn diagram was produced using Venny software [20]. The OR of combinations of risk factors with significant overlap was calculated using binary logistic regression.

For the multivariate logistic, binary logistic and linear regressions a two tailed *p*-value < 0.05 was applied to demonstrate statistical significance. All statistical analyses were performed using STATA (Version 12, StataCorp, College Station, TX).

## Results

**Fig. 1:**
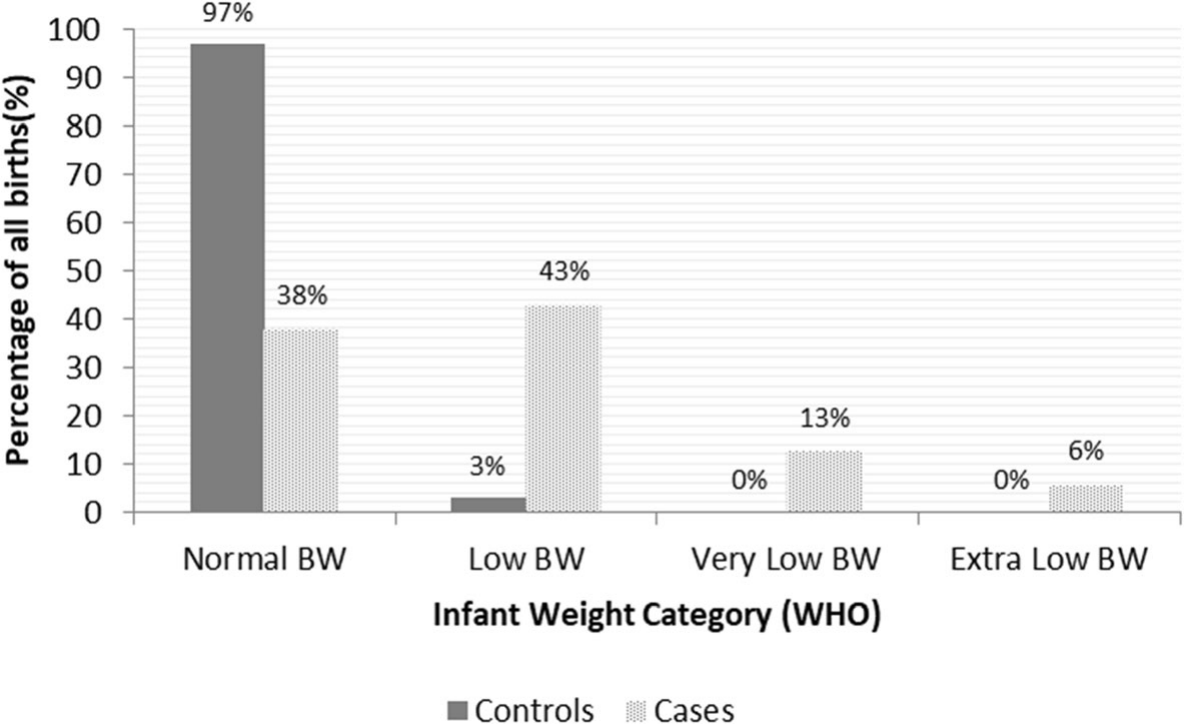
Distribution of infant weight in births from cases and controls. Distribution of infant weight (according to WHO categorization) among births from cases (preterm pregnancies) and controls (term pregnancies). BW: Birth Weight

A total of 697 mothers were included in the study (348 cases and 349 controls). Only one mother did not complete the questionnaire resulting in a final response rate of 99.8% for cases and 100% for controls. Cases were older than controls (30.7, 95% CI: 19.4–41.9 vs 28.8, 95% CI: 18.8–38.7, *p*_value_ < 0.001) and more frequently reported practicing a manual labour profession (33.3% vs 19.8%, *p*_value_ = 0.001) or working > 8 h per day (34.9% vs 11.6%, *p*_value_ < 0.001). Birth weight was significantly lower in cases (mean: 2173 g, 95% CI: 829–3277 among cases Vs mean: 3225 g, 95% CI: 2433–4148 among controls, *p*_value_ < 0.001). The distribution of birth weight in the two groups according to the WHO categorisation is displayed in Fig. 1.

Table 1 presents basic demographic and clinical characteristics of cases and controls. Between the two groups, there were statistically significant differences in the distributions of maternal age, family status, manual labour, long working hours, additional weight gain during pregnancy, pre-gestation BMI, gestational diabetes, depression and stress during pregnancy. Multivariate analysis demonstrated that significant maternal risk factors for prematurity were age (OR: 1.12,95% CI: 1.06–1.18, *p*_value_ < 0.001), gestational diabetes (OR: 0.53,95% CI: 0.30–0.97, *p*_value_ = 0.038), stress (OR: 8.5, 95% CI: 3.03–23.89, p_value_ < 0.001) and long working hours (OR: 3.77, 95% CI: 2.08–6.84, *p*_value_ < 0.001). The results of multivariate analysis for prematurity are summarized in Table 2.

**Table 1:** Distribution of demographic characteristics and maternal risk factors for prematurity among mothers with term (controls) and mother with pre-term deliveries (cases)

Maternal characteristic	Controls *N* (%)	Cases *N* (%)	Statistical Significance†
Demographic			
Age at childbirth^a^	28.8 (18.8–38.7)	30.7 (19.4–41.9)	< 0.001‡
Family status			
Married	339/341 (98.3%)	319/341 (93.6%)	
Divorced	0/341 (0%)	4/341 (1.2%)	0.001
Single	4/341 (1.2%)	18/341 (5.28%)	*P*_trend_: < 0.001
Education level			
Primary	35/343 (10.2%)	36/341 (10.6%)	
Secondary	107/343 (31.2%)	110/341 (32.3%)	
Post-secondary	70/343 (20.4%)	73/341 (21.4%)	0.02
Tertiary	116/343 (33.8%)	87/341 (25.5%)	
Post-graduate	15/343 (4.4%)	35/341 (10.3%)	*P*_trend_: 0.85
Working Conditions			
Unemployed	77/307 (25.1%)	89/320 (27.8%)	0.44
Partner unemployed	64/338 (18.9%)`	54/321 (16.8%)	0.48
Manual labour	46/232 (19.8%)	76/228 (33.3%)	0.001
Prolonged standing at work	103/232(44.4%)	112/229 (48.9%)	0.33
Working > 8 h per day	27/232 (11.6%)	80/229 (34.9%)	< 0.001
Life-Style			
Smoking before pregnancy	92/340 (27.1%)	97/339 (28.6%)	0.65
Smoking during pregnancy	35/341 (10.3%)	38/339 (11.2%)	0.69
Clinical			
Additional weight gain during pregnancy^a^	12.23 (0.3–23.7)	10.96 (3–24)	0.003‡
Body mass index			
BMI < 20	56/334 (16.8%)	65/279 (23.3%)	
20 < BMI < 25	166/334 (49.7%)	132/279 (47.3%)	
25 < BMI < 30	66/334 (19.8%)	55/279 (19.7%)	0.23
30 < BMI < 35	31/334 (9.3%)	19/279 (6.8%)	
BMI > 35	15/334 (4.5%)	8/279 (2.9%)	*P*_trend_: 0.04
Gestational diabetes	74/341 (21.7%)	44/338 (13.0%)	0.003
Clinically diagnosed depression	5/339 (1.5%)	12/339 (3.5%)	0.09
Stress during gestation	12/341 (3.5%)	64/337 (19.0%)	< 0.001

^a^Mean and 95% Confidence Interval

†Independent Sample T test ‡ Pearson Chi Square

**Table 2:** Association between maternal risk factors and prematurity in multivariate analysis

Risk Factor	Contrast	Odds Ratio (95% CI)	*P*-value
Age at child birth	Continuous	1.12 (1.06, 1.18)	< 0.001
Pre-gestation BMI	Continuous	0.96 (0.92, 1.01)	0.11
Gestational diabetes	Categorical	0.53 (0.30, 0.97)	0.04
Depression	Categorical	1.38 (0.25, 7.61)	0.71
Stress	Categorical	8.5 (3.03, 23.89)	< 0.001
Family status	Categorical	1.11 (0.57, 2.15)	0.77
Manual labour	Categorical	1.54 (0.90, 2.65)	0.11
Long working hours	Categorical	3.77 (2.08, 6.84)	< 0.001

**Fig. 2:**
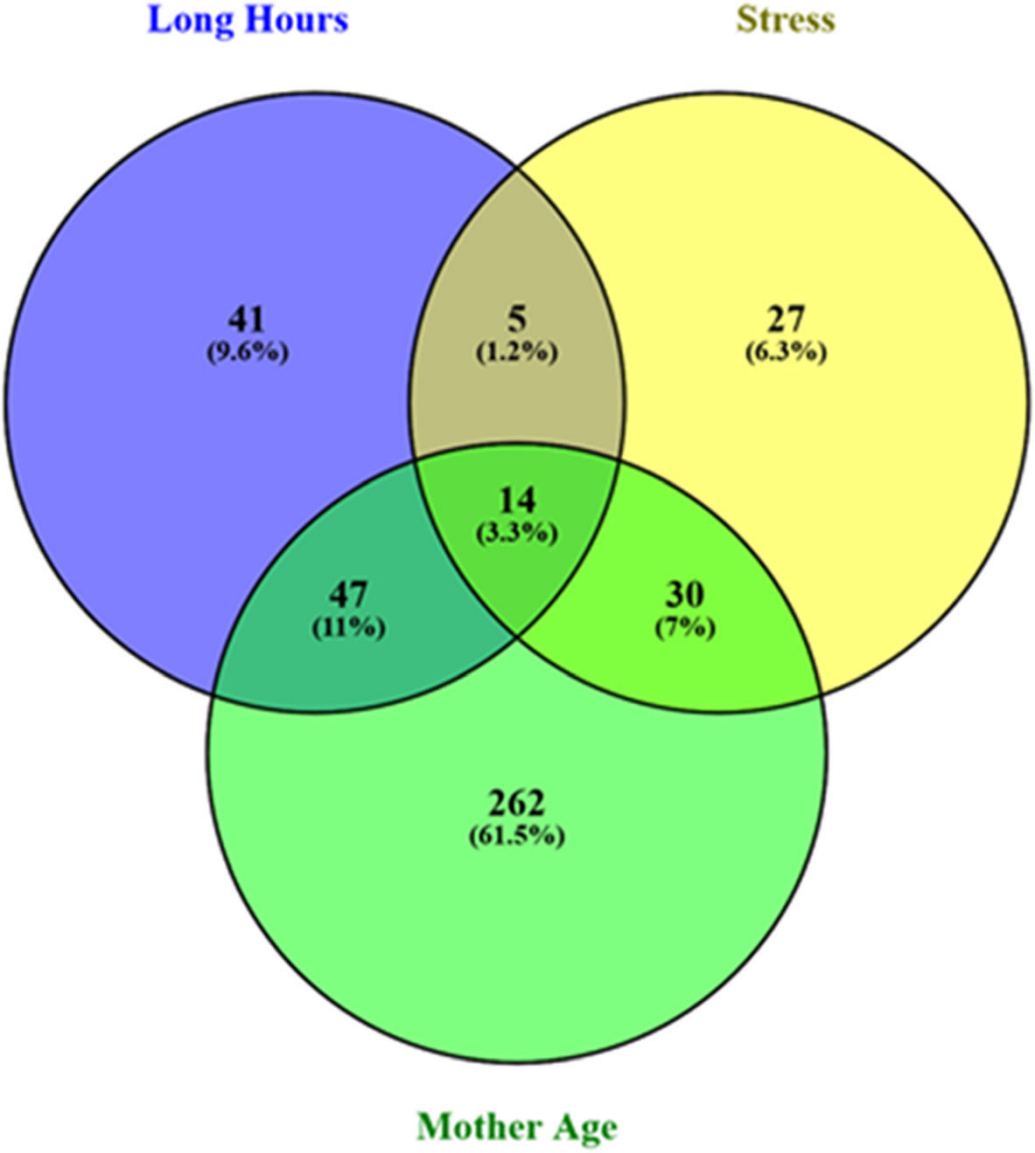
Overlap of significant risk factors for prematurity. Overlap of risk factors that were found to be significant predictors of prematurity (stress, long working hours, mother age > 30 years)

Among the significant maternal risk factors for prematurity, clustering of two or more factors was associated with a sharp increase of prematurity risk as displayed in Table 3. Compared to baseline (no risk factor), the OR for the presence of any one risk factor was 2.37 (95% CI: 1.69–3.32, *p*_value_ < 0.001), the OR for the presence of any two risk factors was 6.13 (95% CI: 3.48–10.80, *p*_value_ < 0.001) and the OR for the presence for any three risk factors was 25.70 (95% CI: 3.31–199.70, *p*_value_ = 0.002). Trend test calculation was statistically significant (*p*_value_ for trend< 0.001). Figure 2, presents the different combinations of significant maternal risk factors in a 3-way Venn diagram. The most frequent combination was advanced maternal age and long working hours (OR: 5.17, 95% CI: 2.60–10.30, *p*_value_ < 0.001) followed by the combination of advanced maternal age and stress (OR: 9.89, 95% CI:3.66–26.72, *p*_value_ < 0.001).

Multivariate analysis within the group of preterm infants, demonstrated that stress was the only parameter that was significantly associated with birth weight (*β*: -323.68, 95% CI: -570.36, − 77.00, *p*_value_ = 0.010). The remaining parameters were not found to significant predictors of birth weight among preterm infants. Table 4 summarizes the results of multivariate analyses for gestational age and infant birth weight within the cases.

**Table 3:** Combined score and associated risk for prematurity

Risk Score^a^	Total Number	Controls (*N* = 343)	Cases (*N* = 342)	OR (95% CI)	*P*-value
0	259	172	87	–	–
1	330	150	180	2.37 (1.69–3.32)	< 0.001
2	82	20	62	6.13 (3.48–10.80)	< 0.001
3	14	1	13	25.70 (3.31–199.70)	0.002

^a^The cumulative individual risk score for each mother was calculated as the sum of values (0 for negative or 1 for positive) for each significant risk factor (long working hours, stress and maternal age). Maternal age was classified as 1 for values ≥30 years old and as 0 for values < 30 years old (30 years was the median value for mother age at childbirth in our dataset)

**Table 4:** Association between maternal risk factors and birth weight within premature infants group

Risk Factor	Contrast	Coefficient (95% CI)	*P*-value
Age at child birth	Continuous	−16.00 (−33.52, 1.54)	0.07
Pre-gestation BMI	Continuous	13.37 (−8.43, 35.16)	0.23
Gestational diabetes	Categorical	201.13 (−69.37, 471.64)	0.14
Depression	Categorical	−34.81 (− 647.09, 577.46)	0.91
Stress	Categorical	−323.68 (−570.36, −77.00)	0.01
Family status	Categorical	145.16 (−66.15, 356.46)	0.18
Manual labour	Categorical	− 154.636 (− 362.05, 52.77)	0.14
Long working hours	Categorical	123.25 (−80.72, 327.22)	0.24

## Discussion

This is the first study conducted in Cyprus, which investigates socioeconomic risk factors for spontaneous premature labor and low birth weight. Our results indicate that advanced maternal age during childbirth, maternal stress and working conditions, are important predictors for preterm delivery and low birth weight.

During the past three decades, an increase in the average childbearing age has been observed among women in high income countries. In the European Union, the average childbearing age was 29.8 years in 2009, compared to 29.3 in 2003 [21] while a similar trend was observed in Australia [22], Canada [23] and the United Kingdom [24, 25]. In 2016, the mean age of mothers at the first childbirth varied between the European Union Member States. The lowest mean age for the first childbirth was recorded in Bulgaria (26.0 years), followed by Romania (26.4), Latvia (26.8), Slovakia (27.0), Poland (27.2) and Lithuania (27.3). In contrast, the mother’s age for the first childbirth was above 30 in Italy (31.0 years), Spain (30.8), Luxembourg (30.5), Greece (30.3) and Ireland (30.1) [26]. In Cyprus, the average childbearing age has increased from 28 to 30.2 years between 1995 and 2015 [23]. Our results are in line with previous findings demonstrating that advanced childbearing age, is a risk factor for premature birth, low birth weight and for adverse pregnancy outcomes such as fetal distress and emergency caesarean section [22, 27, 28]. The increased risk is partly explained by the co-existence of conditions such as diabetes and hypertension [22] and the natural ageing of reproductive tissues which may result in reduced fetal intake of necessary nutrients for intrauterine growth [29].

Unfavorable working conditions, characterised by manual labour and long working hours in particular, were associated with preterm birth among Cypriot women in this study. Previous findings have also demonstrated a positive association of physical exertion related work, long hours and/or shift work with poor pregnancy outcomes [30, 31]. These effects can be attributed to muscles physical stress at work and increased release of catecholamines and arteriol constriction, which causes redistribution of blood flow in the pregnant woman and reduced blood flow to the placenta, as well as hormonal disturbances and nutritional deficits that can also adversely affect fetal growth [32]. Overall, although there is considerable inhomogeneity in the employment settings among the published studies that explore the relationship between working conditions and pregnancy outcomes [33], it is now well documented that long working hours constitute a significant risk factor for premature birth and low birth weight [34, 35].

Maternal stress during pregnancy was also a statistically significant predictor in the occurrence of both preterm birth and low birth weight in our study. Despite the heterogeneity of previous studies’ design and approaches to measuring stress, published literature indicates a strong association between stress during pregnancy and risks for premature birth and low birth weight [36, 37]. In a recent Swedish study more than 50% of pregnant women reporting stress during pregnancy experienced a premature labor [37], while other studies besides preterm birth associated stress with low birth weight and low head circumference [38, 39]. As a consequence, it is necessary for pregnant women to be monitored regularly to detect development of stress and other psychological problems [40]. According to the guidelines of the American College of Obstetricians and Gynaecologists, it is recommended that pregnant women are screened at least once every trimester during pregnancy for their psychological condition, irrespectively of the social and educational level, race and ethnicity, and referred for specialised treatment if applicable [40, 41].

In general, gestational diabetes mellitus has been found to be associated with medically indicated premature labor and lower gestational age [42, 43]. However, in our study, we found that the frequency of gestational diabetes was lower in mothers who had premature birth compared to controls. Similar negative associations between pregnancy outcomes and gestational diabetes have been also reported by few recent studies [44, 45]. These discrepancies can be attributed to the possibility of good glycemic control of women with gestational diabetes in these studies through good obstetric monitoring, balanced diet and insulin treatment, factors which have not been specifically assessed in our study [46, 47]. A recent study demonstrated that although presence of uncontrolled gestational diabetes and obesity during pregnancy is associated with negative prognosis, their effects can be counterbalanced by the application of glycaemic control combined with controlled weight gain [48]. Furthermore, comparison with previous studies is inherently difficult as the effect of gestational diabetes on perinatal outcomes is influenced by racial factors [49], the different diagnostic criteria for gestational diabetes that are used in each country, the heterogeneity of study populations and differences in the detection programs that are applied in each country, which eventually result in a wide range of gestational diabetes frequency from less than 1% to above 10% across the world [46].

This study benefited largely by including data from consecutive births in Cyprus, accurate assessment of study outcomes, a very high response rate and a questionnaire that captured responses on a wide array of socioeconomic factors. However, our study has several limitations, primarily originating from its retrospective nature which precluded the acquisition of participants’ detailed clinical data such as obstetric complications [50, 51], use of corticosteroids [52] and other medications that may affect occurrence of premature birth [28] and the type and quality of the provided obstetric care [53]. Like any questionnaire study, this study might have been influenced by subjectivity and recall bias, although factors like family status, working conditions and smoking habits during pregnancy are usually easily recollected characteristics by mothers. Furthermore, assessment of factors, such as emotional stress with the use of a one-time questionnaire during the postpartum period that was not specifically designed to address psychological parameters, might have also introduced some bias in our results [54]. Future studies, need to further explore the findings of the present study in the Cyprus population with a prospective study design and the use of validated instruments for measurement of mental health attributes [55–57]. Furthermore, our study excluded women with multiple pregnancies or infertile women who underwent infertility treatment. As a result, the generalizability of our findings is limited to women with singleton pregnancies following natural conception.

## Conclusions

In summary, stress, prolonged working hours and advanced maternal age at childbirth, are associated with increased odds of preterm delivery and low birth weight in Cyprus, while the combination of adverse socioeconomic risk factors appears to have a cumulative effect on the odds of prematurity. Further, prospective, studies should further investigate risk factors for adverse pregnancy outcomes and eventually inform local public health authorities towards the development of evidence-based management protocols to limit premature births and subsequent neonatal complications and related healthcare costs.

## Additional file

Additional file 1: Study Questionnaire in English language. (DOCX 24 kb)

## Abbreviations

BMI: Body Mass Index
CI: Confidence Intervals
LBW: Low Birth Weight
NICU: Neonatal Intensive Care Unit
OR: Odds Ratio
WHO: World Health Organisation
