# Supplementary material for: Maternal socioeconomic factors and the risk of premature birth and low birth weight in Cyprus: a case–control study
Source: Reprod Health. 2018 Sep 19;15:157. doi: 10.1186/s12978-018-0603-7 (PMC6146509; doi:10.1186/s12978-018-0603-7)
Supplement: Supplementary file 1 — Study Questionnaire in English language. (DOCX 24 kb) [file 12978_2018_603_MOESM1_ESM.docx]

**QUESTIONNAIRE**

SOCIO-ECONOMIC CHARACTERISTICS & PREMATURE BIRTH

THE CASE OF CYPRUS

1. **Age of the mother on the date of the delivery:**
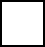

2. **Gestation age in weeks (e.g. 38 weeks):**
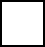

3. **Neonatal birth weight in grams:**
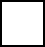


**4. Level of education** [**put it in circle**]

**4.1 Secondary school graduate or less**

**4.2 High school graduate**

**4.3 College or Professional School graduate**

**4.4 University graduate**

**4.5 Doctorate or Master’s program graduate**

**5. Ethnicity** [**put it in circle**]

**5.1 Greek Cypriot**

**5.2 Other (please specify): ………………………………..**

**6. Work conditions**

**6.1 Is your work manual; Yes
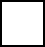
 No
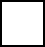
**

**6.2 Do you stand a lot of hours during work; Yes
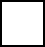
 No
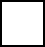
**

**6.3 Do you work more than 8 hours a day; Yes
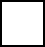
 No
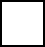
**

**7. Family status: [put it in circle]**

**7.1 Married**

**7.2 Divorced**

**7.3 Widowed**

**7.4 Single parent**

**8. Income status:**

**8.1 Are you unemployed; Yes
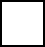
 No
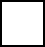
**

**8.2 Is your husband unemployed; Yes
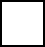
 No
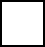
**

**9. Do you have free access to the national public health system**

**Yes**
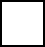
 **No**
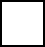


**10. Medical history during pregnancy:**

**10.1 How many kilograms have you gained during pregnancy:
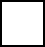
**

**10.2 Weight before pregnancy:
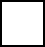
 Kg, and height before pregnancy:
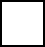
 cm.**

**10.3 Diabetes during pregnancy: Yes
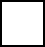
 No
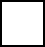
**

**10.4 Smoking during pregnancy: Yes
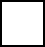
 No
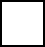
**

**10.5 If yes, how many cigarettes per day: ………**

**10.6 Smoking before pregnancy: Yes
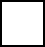
 No
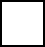
**

**10.7 If yes, how many cigarettes per day: ………**

**10.8 Alcohol consumption during pregnancy: Yes
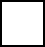
 No
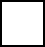
**

**10.9 Physician diagnosis of depression during pregnancy:**

**Yes
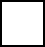
 No
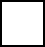
**

**10.10 Have you experienced any emotional stress or anxiety during pregnancy;
 Yes
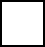
 No
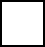
**

**10.11 Were you prescribed any antidepressant medication during pregnancy:
 Yes
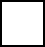
 No
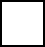
**
